# Current Pipelines for Neglected Diseases

**DOI:** 10.1371/journal.pntd.0003092

**Published:** 2014-09-04

**Authors:** Paolo di Procolo, Claudio Jommi

**Affiliations:** 1 Department of Pharmaceutical Sciences, Università del Piemonte Orientale, Novara, Italy; 2 CERGAS (Centre for Research on Health and Social Care Management), Università Bocconi, Milano, Italy; The George Washington University Medical Center, United States of America

## Abstract

This paper scrutinises pipelines for Neglected Diseases (NDs), through freely accessible and at-least-weekly updated trials databases. It updates to 2012 data provided by recent publications, and integrates these analyses with information on location of trials coordinators and patients recruitment status. Additionally, it provides (i) disease-specific information to better understand the rational of investments in NDs, (ii) yearly data, to understand the investment trends. The search identified 650 clinical studies. Leishmaniasis, Arbovirus infection, and Dengue are the top three diseases by number of clinical studies. Disease diffusion risk seems to be the most important driver of the clinical trials target choice, whereas the role played by disease prevalence and unmet need is controversial. Number of trials is stable between 2005 and 2010, with an increase in the last two years. Patient recruitment was completed for most studies (57.6%), and Phases II and III account for 35% and 28% of trials, respectively. The primary purpose of clinical investigations is prevention (49.3%), especially for infectious diseases with mosquitoes and sand flies as the vector, and treatment (43.2%), which is the primary target for parasitic diseases Research centres and public organisations are the most important clinical studies sponsors (58.9%), followed by the pharmaceutical industry (24.1%), foundations and non-governmental organisations (9.3%). Many coordinator centres are located in less affluent countries (43.7%), whereas OECD countries and BRICS account for 34.7% and 17.5% of trials, respectively. Information was partially missing for some parameters. Notwithstanding, and despite its descriptive nature, this research has enhanced the evidence of the literature on pipelines for NDs. Future contributions may further investigate whether trials metrics are consistent with the characteristics of the interested countries and the explicative variables of trials location, target (disease) choice, and the object of the trials.

## Author Summary

Neglected diseases lead to illness, long-term disability and affect economic development in poor populations. There is evidence that clinical research on neglected diseases has increased starting from the second half of the '90s. This paper aims at updating this evidence to 2012 and at integrating available data (groups of target of the clinical projects, phase in the clinical development process, sponsors) with other data that have not been investigated or published so far (recruitment status of patients, and trials location of the trials coordinator). Our study has confirmed previous findings on the important investment in NDs, highlighting, thanks to a disease-specific approach, a particular focus on diseases with a higher diffusion risk, but not necessarily the higher prevalence and the most unmet need. In most studies, patients' recruitment has been completed, and many trials are also in the very terminal phases: this means a high probability that new treatments will be available in the next years. In addition, trial coordinator centres are increasingly located in low income countries; as a consequence, the investment in clinical research has become an opportunity to further enhance clinical and organisational expertise in these countries.

## Introduction

Neglected diseases (NDs) may be defined as ancient, disabling, and poverty-promoting chronic conditions that afflict the poorest people in the developing world [1]. These diseases represent the most widespread viral, parasitic, and bacterial infections in those countries with people living on less than US $ 2 per day [2]. NDs can lead to long-term disability and poverty, as a result of impaired childhood growth and development, adverse outcome of pregnancy, and reduced productive capacity.

There is not a unique list of NDs. The World Health Organization (WHO) [3] defines “neglected” as the 17 “…chronically endemic and epidemic-prone tropical diseases, which have a very significant negative impact on the lives of poor populations [and] remain critically neglected in the global public health agenda”. According to the Public Library of Science for Neglected Tropical Diseases (PLoS NTD) [4], NDs “[neglected tropical diseases] are defined as a group of poverty-promoting chronic infectious diseases, which primarily occur in rural areas and poor urban areas of low-income and middle-income countries. They are poverty-promoting because of their impact on child health and development, pregnancy, and worker productivity, as well as their stigmatizing features”. Merging the lists suggested by the WHO and PLoS NTD, more than 40 NDs were listed (see Box 1).

**Box 1. List of NDs considered**

WHO NDs list

Ascariasis, Buruli Ulcer, Chagas Disease, Cysticercosis/teniasis, Dengue/dengue haemorrhagic fever, Dracunculiasis (guinea-worm disease), Echinococcosis, Food-borne Trematodiases, Fascioliasis, Hookworm Infections, Human African Trypanosomiasis, Leishmaniasis, Leprosy, Lymphatic Filariasis, Onchocerciasis, Rabies, Schistosomiasis, Soil transmitted Helminthiasis, Trachoma, Trichuriasis, Yaws, Treponematosis (Bejel, Pinta, Syphilis).

Diarrhoeal Diseases

Amebiasis, Cholera, Enteric pathogens (Shigella, Salmonella, E.coli), Giardiasis.

Other NDs

Balantidiasis, Bovine Tuberculosis in Humans, Other arboviral infections, Bartonella, Loiasis, Mycetoma, Myiasis, Paracoccidioidomycosis, Relapsing Fever, Scabies, Strongyloidiasis, , Toxocariasis and other Larva Migrans, Viral hemorrhagic fever, Yellow Fever

Table 1 illustrates the prevalence, mortality rates, and current treatment for some NDs. Precise epidemiological data are not available for all NDs. In fact, some of them are either endemic in the poorest and most rural world areas or difficult to diagnose. Soil-transmitted Helminthiasis, Schistosomiasis, Lymphatic filariasis, Trachoma, Dengue, Onchocerciasis, and Leishmaniasis are the most common NDs. They are mostly caused by parasites, poor sanitation, and other environmental factors. Their current treatments, if any, show poor effectiveness (e.g., the longer is the exposure to Chagas disease before treatment, the lower is the effectiveness of the combination of benznidazole and nifurtimox) and/or important side effects.

**Table 1:** Burden and current treatment of major NDs.

Disease	Prevalence (per one million)	Annual Death	Current treatment	Reference
**Buruli Ulcer**	0.05	Unknown	Bacille Calmette-Guérin (BCG) vaccine, Combination of rifampicin and streptomycin/amikacin, Surgery to remove necrotic tissue, cover skin defects and correct deformities	[16]
**Chagas disease**	8.5	14000	Benznidazole and nifurtimox	[16]
**Dengue**	50	12500	Maintenance of the patients circulating fluid volume	[16]
**Dracunculiasis**	0.01	Unknown	Manual extraction of worm	[16]
**Echinococcosis**	Unknown	Unknown	Surgical intervention or percutaneous treatment and/or high dose, long-term therapy with albendazole alone or in combination with praziquantel	[16]
**Foodborne trematode infections**	56	7000	Triclabendazole and praziquantel	[17]
**Human African trypanosomiasis**	0.3	48000	Pentamidine, suramin, melarsoprol and eflornithine. Pentamidine and suramin are used in the first or early stage of T.b.gambiense and T.b. rhodesiense infections respectively. Melarsoprol is used in the second or advanced stage of both forms of the disease, being the only treatment available for late stage of T.b. rhodesiense. Eflornithine can be used in monotherapy but only in the second stage of the T.b.gambiense infections.	[16]
**Leishmaniasis**	12	51000	Amphotericin B, liposomal amphotericin B, miltefosine, paromomycin, sodium stibogluconate, WHO-approved generic, sodium stibogluconate, meglumine antimoniate	[16]
**Leprosy**	0.34	6000	Multi drug therapy: a combination of rifampicin, clofazimine and dapsone and rifampicin and dapsone	[16]
**Lymphatic filariasis**	120	<500	Diethylcarbamazine citrate (DEC) + albendazole; or 150 µg/kg of body weight ivermectin + albendazole (in areas that are also endemic for onchocerciasis).	[16]
**Onchocerciasis**	37	<500	Ivermectine	[16]
**Rabies**	Unknown	Unknown	Pre-exposure prophylaxis: cell culture-based vaccines with periodic booster injections Post-exposure prophylaxis: washing and flushing with soap/detergent and copious amounts of water	[18] [19]
**Schistosomiasis**	207	150000–200000	Preventive chemotherapy, mass treatment without individual diagnosis or praziquantel	[16]
**Soil-transmitted helminths Ascariasis (roundworm)**	807	3000–60000	Albendazole, mebendazole, praziquantel	[16]
**Soil-transmitted helminths Trichuriasis (whipworm)**	604	3000–10000	Albendazole, mebendazole, praziquantel	[16]
**Soil-transmitted helminths Hookworm**	576	3000–65000	Albendazole, Mebendazole, Praziquantel	[16]
**Cysticercosis Taeniasis**	Unknown	Unknown	Preventive chemotherapy, mass treatment without individual diagnosis, praziquantel or niclosamide	[20]
**Trachoma**	84	<500	Surgery, antibiotic treatment	[16]
**Yaws**	Unknown	Unknown	Azithromycin, benzathine, penicillin	[21]

These diseases were given low priority by the pharmaceutical industry and other actors before the new millennium. According to a Wellcome Trust Report [5], only 13 out of 1,393 new drugs developed during 1975 to 1999 were for NDs.

However, after the 2000 WHO initiative on Millennium Development Goals (MDGs), the international health policy agenda put NDs in high consideration [6]. At the same time, the industry has started to include public health objectives in their ethical responsibilities and other sectors (governments, non-governmental organisations [NGOs], and international health organisations) have begun to look at the private sector as a partner. This new scenario fostered the development of Public-Private Partnerships (PPPs), because joining the strengths and skills of the two parties seemed a feasible and effective way to tackle complicated and expensive public health problems [7]. Incentives and PPPs increased investments in NDs, with more than 60 projects in progress at the end of 2004 [5]. HIV/AIDS, tuberculosis and malaria were the primary diseases addressed by global fund and health interventions for all NDs [8].

After 2005, the WHO, NGOs and foundations recognised the lack of effective global prevention and control programs to overcome NDs. WHO created the Global Plan to combat NDs. The goal of the Global Plan was to prevent, control, eliminate or eradicate NDs by 2015 [9]. However, the Global Plan did not achieve the expected goals, and the deadline to prevent and control programs was postponed to 2020 [10]. The literature has further tracked the increase in investments in R&S for NDs. Under the umbrella of the G-FINDER project, a report has investigated the amount of money invested in projects on NDs [11]. Bio Ventures for Global Health [12] has collected data on pipelines for NDs from multi-sources, including websites and reports, press releases and scientific literature, and clinical trials databases. The most recent contribution has investigated both products approved in 2000–2011 and pipelines for NDs (derived from the NIH – National Institute for Health and WHO databases) as of December 2011, showing that NDs (including malaria and tuberculosis) account for 4% of total products launched into the market in 2000–2011 and 1% of pipelines as of December 2011 [13].

This evidence has produced new important information on investments in NDs. However not all diseases listed into the Box 1 have been covered. Additionally, the latest and most complete analysis does not provide disease-specific data. These data may be useful to understand the drivers of investments allocation. Location of trials coordinator and patients enrolment status have not been investigated or reported in most of these studies. Finally, the evolution of pipelines in time has not been considered. Our objective is to cover these information gaps and update to pipelines analysis to 2012.

## Materials and Methods

The list of NDs investigated resulted from the merging of the WHO and PLoS NTD lists (see Box 1). The disease or group names (e.g., Arbovirus, Hookworm, and Enteric pathogens) were used to extract the relevant trials from the databases. We have excluded malaria and tuberculosis. They are not included into the WHO and PLOS lists of NDs and the investment in these diseases have been compared with other NDs by other authors [11].

The following access-free and at-least-weekly updated trial databases were considered: the U.S. clinical trial database (http://www.clinicaltrials.gov/), the European clinical trial database (https://www.clinicaltrialsregister.eu/), the International Standard Randomised Controlled Trial Number Register (http://www.controlled-trials.com/isrctn/), the Indian clinical trial database (http://ctri.nic.in/Clinicaltrials/login.php), and the Australian clinical trial database (http://www.anzctr.org.au/). Other registries, included in the WHO list (http://www.who.int/ictrp/network/primary/en/index.html), were not included in the search strategy because very few trials were extracted and most of them matched what has been found using the above-mentioned databases. In principle, the WHO clinical trial database merges the information of all trial databases, but extracting information from primary databases was preferred to be sure that the most recent trials were included. All trials databases have been accessed last time December, 31st, 2012.

Trials received from January 1^st^ 2005 to December 31^st^ 2012 were extracted for each disease listed in Box 1. The following inclusion criteria were used:

- only interventional clinical trials;
- only trials on bacterial, viral or other pathogen-caused diseases in Box 1;
- only trials for which at least one of the following database sections was completed: description of the condition, general information, brief summary, and intervention;
- clinical trials on enteric diseases in Box 1 were included if associated with diarrheal symptoms and excluded if related to urinary tract infection;
- behavioural trials for syphilis were not included even if interventions were conducted.

Clinical trials were classified and analysed according to:

- the disease/indication investigated;
- the trial status: (i) not yet recruiting (patients are not yet being recruited or enrolled); (ii) recruiting (participants are currently being recruited and enrolled); (iii) enrolling by invitation (patients are being selected from a selected target); (iv) withdrawn (study has halted prematurely, prior to enrolment of the first participant); (v) terminated (recruitment or enrolment has halted prematurely and will not start again); (vi) suspended (recruitment or enrolment of participants has halted prematurely but may start again); (vii) completed;
- the study phase: phase I, which generally tests a new drug or treatment in a small group of healthy people to determine the metabolism and pharmacologic actions; phase II, which expands the study to a group of patients with the disease or condition under study to investigate efficacy and determine the common short-term side effects and risks; phase III, which expands the study to a larger group of patients to gather information on the overall risk-benefit; and phase IV, which includes all post-marketing studies;
- the type of intervention investigated: biological; drug; diagnostics/devices; or others, such as procedures and educational and behavioural interventions;
- the primary purpose of the intervention: basic science, screening, prevention, diagnosis, treatment, supportive care and education/counselling/training health service research;
- the location of trials coordinator;
- the sponsor, i.e. the trial promoter that may be different from the trial funder. Sponsors have been classified into three categories: Industry, NGOs and Foundations, Public institutions and Research centres. Organisations where classified as Foundations if this is explicitly declared in the relevant website (general or financial information). Public institutions include both governmental organisations and international organisations.

## Results

The research identified 650 clinical studies. Figures are not comparable with the pipelines for important diseases in affluent countries, e.g., cardiovascular diseases (15,232 clinical trials) or respiratory diseases (10,063 clinical trials). Total number of trials has been rather constant over time, with an important increase in the last two years covered by our analysis (2011–2012). Total number of trials have been rather constant over time, with an important increase in the last two years covered by our analysis (2011–2012). The increase in the last two years is mainly driven by trials of WHO NDs list (Box 1). This trend may explain why we have found a lower number of trials for diarrhoeal diseases in our NDs list, than what have been found as of the end of 2011 by Pedrique and colleagues [13] (Table 2).

**Table 2:** Number of trials per disease groups and year.

	2005	2006	2007	2008	2009	2010	2011	2012	Total	Pedrique et al (2013) (2000–2011)
NDs in WHO List	45	39	33	49	56	48	68	68	406	420
Diarrhoeal diseases	18	14	8	14	15	17	19	15	120	270
Others NDs	23	10	12	20	16	13	18	12	124	158
**Total**	**86**	**63**	**53**	**83**	**87**	**78**	**105**	**95**	**650**	**848**
NDs in WHO List	52.3%	61.9%	62.3%	59.0%	64.4%	61.5%	64.8%	71.6%	62.5%	49.5%
Diarrhoeal diseases	20.9%	22.2%	15.1%	16.9%	17.2%	21.8%	18.1%	15.8%	18.5%	31.8%
Others NDs	26.7%	15.9%	22.6%	24.1%	18.4%	16.7%	17.1%	12.6%	19.1%	18.6%
**Total**	**100.0%**	**100.0%**	**100.0%**	**100.0%**	**100.0%**	**100.0%**	**100.0%**	**100.0%**	**100.0%**	**100.0%**

**Figure 1:**
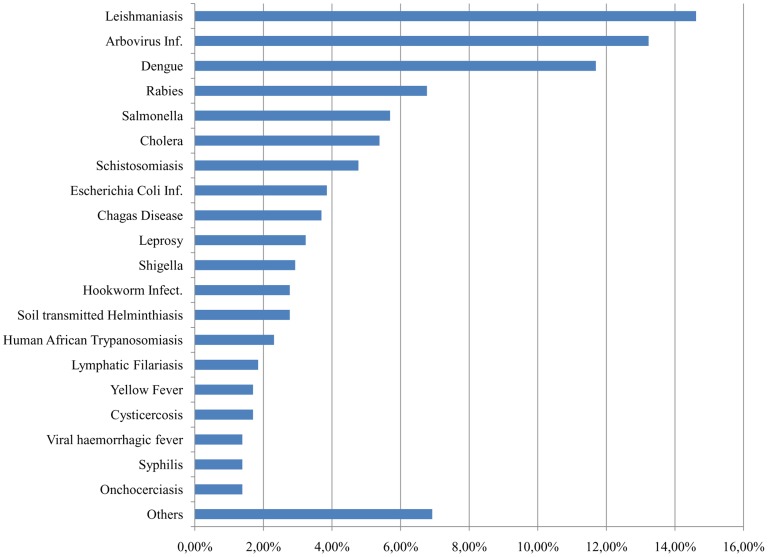
Interventional clinical trials for NDs: distribution per indication.

Leishmaniasis (95 studies), arthropod-borne viruses (Arbovirus) infection (86 studies), and Dengue (76 studies) are the top three diseases by number of clinical studies (Figure 1). These three diseases represent almost 50% of all NDs studies, followed by enteric diseases (Salmonella, Cholera, Shigella and *Escherichia coli* infection), which cumulatively account for 18% of total trials. The group “other diseases” (diseases with less than 9 trials) includes, among others, Buruli Ulcer and Ascariasis, which are recognised as severe diseases.

**Figure 2:**
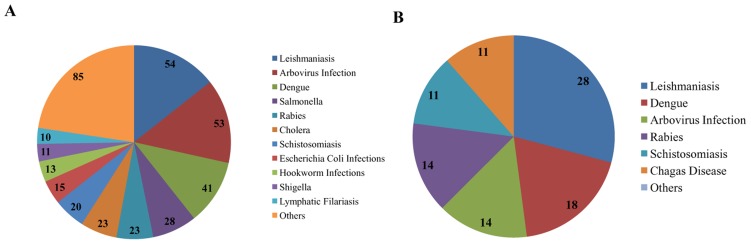
(A) Interventional clinical trials for NDs: analysis of completed trials and (B) those recruiting patients.

For most of the 650 trials, recruitment of patients is completed (57.6%) (Figure 2). The patient recruitment process is ongoing for 24.9% of trials. Only 12.5% of the studies are either temporarily or definitely suspended and the trial status is unknown for 5.1% of the studies. Leishmaniasis (54 studies), Arbovirus infections (53 studies) and Dengue (41 studies) again show the largest number of completed trials. These three diseases are also the object of the highest number of trials in which patients have been enrolling.

The distribution of clinical studies per development phase is strongly affected by a huge proportion (24.9%) of missing data and trials allocated between phase I and II or phase II and III (6%) (Table 3). For some diseases (i.e., Schistosomiasis, Leprosy, soil-transmitted Helminthiasis), the number of trials missing information on the trial phase exceeds the number of trials where the phase is specified. Considering trials allocated to a single phase, 100 (22%) are in phase I, 135 (35%) in phase II, 125 (28%) in phase III and 88 (20%) in phase IV. Dengue and Leishmaniasis account for 37.5% of trials in early phases (I and II), whereas Arbovirus infections, Leishmaniasis and Rabies represent 47% of clinical trials in phase III. Phase IV studies are more frequent in Arbovirus infections, Leishmaniasis and Salmonella.

**Table 3:** Distribution of interventional clinical trials for NDs per phase.

Disease	Number						%*			
	Phase I	Phase II	Phase III	Phase IV	N.A	Total	Phase I	Phase II	Phase III	Phase IV
Leishmaniasis	10	30	17	10	28	95	14.9%	44.8%	25.4%	14.9%
Arbovirus Infection	8	23	31	21	3	86	9.6%	27.7%	37.3%	25.3%
Dengue	29	18	8	-	21	76	52.7%	32.7%	14.5%	-
Rabies	4	9	15	7	9	44	11.4%	25.7%	42.9%	20.0%
Salmonella	4	11	5	10	7	37	13.3%	36.7%	16.7%	33.3%
Cholera	3	12	8	5	7	35	10.7%	42.9%	28.6%	17.9%
Schistosomiasis	2	3	6	3	17	31	14.3%	21.4%	42.9%	21.4%
Escherichia Coli Infections	9	4	4	1	7	25	50.0%	22.2%	22.2%	5.6%
Chagas Disease	-	4	4	9	7	24	-	23.5%	23.5%	52.9%
Leprosy	1	4	2	4	10	21	9.1%	36.4%	18.2%	36.4%
Shigella	6	5	3	-	5	19	42.9%	35.7%	21.4%	-
Soil transmitted Helminthiasis	3	-	1	4	10	18	37.5%	-	12.5%	50.0%
Hookworm Infections	3	-	-	3	12	18	50.0%	-	-	50.0%
Human African Trypanosomiasis	4	2	3	1	5	15	40.0%	20.0%	30.0%	10.0%
Lymphatic Filariasis	-	1	-	-	11	12	-	100.0%	-	-
Yellow Fever	2	2	3	1	3	11	25.0%	25.0%	37.5%	12.5%
Cysticercosis	3	-	3	-	5	11	50.0%	-	50.0%	-
Onchocerciasis	-	1	1	-	7	9	-	-	-	-
Syphilis	1	2	4	-	2	9	14.3%	28.6%	57.1%	-
Viral haemorrhagic fever	8	1	-	-	-	9	-	-	-	-
Others	0	3	8	9	25	45	0	15.0%	40.0%	45.0%
**Total**	**100**	**135**	**126**	**88**	**201**	**650**	**22.3%**	**30.1%**	**28.1%**	**19.6%**

* Net of trials where the phase is not specified.

The primary purpose of the identified trials is illustrated in Table 4. The greatest proportion of clinical studies have prevention (41.4%; 49.3% if only trials where the purpose is specified are considered) or treatment (185 trials, 36.3%; 43.2% of trials, net of whose purpose is not specified) as the primary purpose. Prevention is the most important target for diseases in which the method of transmission is a vector such as the mosquito or sand fly, including Dengue and Arbovirus infections. For parasitic diseases, such as Leishmaniasis, soil-transmitted Helminthiasis and Chagas disease, most trials have treatment as the primary purpose.

**Table 4:** Primary scope of interventional clinical trials for NDs.

Disease	Number							%*				
	B	P	D+S	T	Others	N.A.	Total	B	P	D+S	T	Others
Leishmaniasis	1	10	1	80	1	2	95	1.1%	10.8%	1.1%	86.0%	1.1%
Arbovirus Infection	5	64	-	8	-	9	86	6.5%	83.1%	-	10.4%	-
Dengue	-	57	-	6	1	12	76	-	89.1%	-	9.4%	1.6%
Rabies	-	28	-	10	2	4	44	-	70.0%	-	25.0%	5.0%
Salmonella	1	24	1	4	1	6	37	3.2%	77.4%	3.2%	12.9%	3.2%
Cholera	1	19	-	6	3	6	35	3.4%	65.5%	-	20.7%	10.3%
Schistosomiasis	-	7	-	14	-	10	31	-	33.3%	-	66.7%	-
Escherichia Coli Infections	1	14	-	5	-	5	25	5.0%	70.0%	-	25.0%	-
Chagas Disease	1		1	17	1	4	24	5.0%	0.0%	5.0%	85.0%	5.0%
Leprosy	-	3	1	13	-	4	21	-	17.6%	5.9%	76.5%	-
Shigella	1	14	-	2	-	2	19	5.9%	82.4%	-	11.8%	-
Soil transmitted Helminthiasis	-	7	-	10	-	1	18	-	41.2%	-	58.8%	-
Hookworm Infections	1	2	2	2	-	11	18	14.3%	28.6%	28.6%	28.6%	-
Human African Trypanosomiasis	1		-	9	-	5	15	10.0%	0.0%	-	90.0%	-
Lymphatic Filariasis	-		-	5	-	7	12	-	0.0%	-	100.0%	-
Cysticercosis	-	2	3	6	-	-	11	-	18%	27%	55%	-
Yellow Fever	2	7	1		1	-	11	18%	64%	9%	-	9%
Onchocerciasis	-		-	1	-	8	9	-	-	-	100.0%	-
Syphilis	-	6	1		1	1	9	-	75.0%	12.5%	-	12.5%
Viral haemorrhagic fever	-	3	-	6	-	-	9	-	33%	-	67%	-
Others	1	2	3	32	-	7	45	2.6%	5.3%	7.9%	84.2%	-
**Total**	**16**	**269**	**14**	**236**	**11**	**104**	**650**	**2.9%**	**49.3%**	**2.6%**	**43.2%**	**2.0%**

* Net of trials where the primary scope is not specified.

BS: Basic science; P: Prevention; D-S: Diagnostic/Screening; T: Treatment; Others: Health service research, Supportive care, Educational/Counselling/Training and Others; N.A: Not Available; Other diseases include Trichuriasis, Strongyloidiasis, Scabies, Trachoma, Ascariasis, Buruli Ulcer, Loiasis, Fascioliasis, Relapsing Fever, Giardiasis, Amebiasis, Echinococcosis, Yaws, Mycetoma, Toxocariasis, Taeniasis, Bartonella, Food-borne Trematodiases.

**Figure 3:**
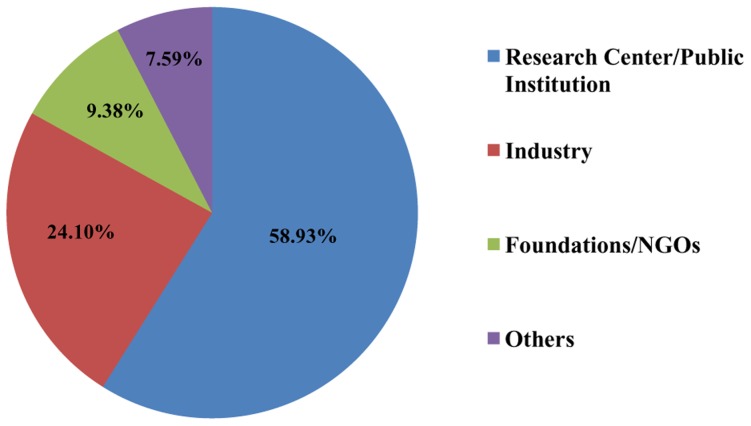
Sponsors of interventional clinical trials for NDs.

Research centres and public organisations are the most important sponsors of clinical studies on NDs (58.9% of studies), followed by the industry (24.1%), foundations and NGOs (9.3%) (Figure 3). These figures are different from what can be found for Research and Development (R&D) on targets prevailing in affluent countries, where the industry plays a major role as sponsor, especially in the pre-marketing phase, and may be also co-funder of non-profit studies. Foundations play a minor role as sponsors of NDs. However, many research centres (inside or outside universities) receive research grants from foundations (e.g., the Wellcome Trust), thus making these groups funders, but not sponsors, of the relevant clinical studies.

The National Institute of Allergy and Infectious Diseases (NIAID) (within the National Institutes of Health) and the U.S. Army Medical Research and Material Command are the most important funders among public organisations and research centres. The huge investment by the U.S. Army Medical Research centre is mainly motivated by the presence of the U.S. Army in low-income countries where NDs are endemic. The target of clinical trials sponsored by the U.S. Army Medical Research and Material Command are Arbovirus infection (9 studies), Dengue (7 studies) and Leishmaniasis (10 studies), because they have a greater potential to cross national borders than other diseases.

Apart from the International Centre for Diarrheal Disease Research, located in Bangladesh, and the International Vaccine Institute, the major sponsors among research centres are all located in the US and the UK.

Sanofi-Aventis is the pharmaceutical company most involved as sponsor in trials for NDs, most of which are related to Arbovirus infections and Dengue, with a particular interest in vaccines and viral treatments. Novartis and Novartis Vaccines, with 22 trials, is the second largest sponsor from the pharmaceutical industry, with a focus on Rabies and Salmonella. Noticeably, many trials on Arbovirus infections are sponsored by Intercell AG, a small biotech company that develops vaccines for the prevention and treatment of infectious diseases (especially Japanese Encephalitis).

The Drugs for Neglected Diseases initiative (DNDi) is certainly the most important NGO involved in trials for NDs, with 15 studies sponsored mostly related to human African Trypanosomiasis and Leishmaniasis.

Other foundations, like Oswaldo Cruz Foundation (6 clinical trials) or the AB Foundation with 5 clinical trials have sponsored trials mostly on Leishmaniasis (Table 5).

**Table 5:** Distribution of trials among sponsor for NDs.

Research centres/Public institutions	Trials
National Institute of Allergy and Infectious Diseases (NIAID)	43
U.S. Army Medical Research and Materiel Command	30
International Centre for Diarrhoeal Disease Research (Bangladesh)	19
International Vaccine Institute	17
London School of Hygiene and Tropical Medicine	11
University of Oxford (UK)	10

Industry	Trials
Sanofi-Aventis	43
Novartis	22
Intercell AG	18
Baxter Healthcare Corporation	16
GlaxoSmithKline	6
Inviragen Inc.	5

Foundations/NGOs	Trials
Drugs for Neglected Diseases initiative DNDi	15
Oswaldo Cruz Foundation	6
Institute of Tropical Medicine (Belgium)	6
AB Foundation	5
Volkswagen Foundation (VolkswagenStiftung) (Germany)	4
Médecins Sans Frontières	2

**Figure 4:**
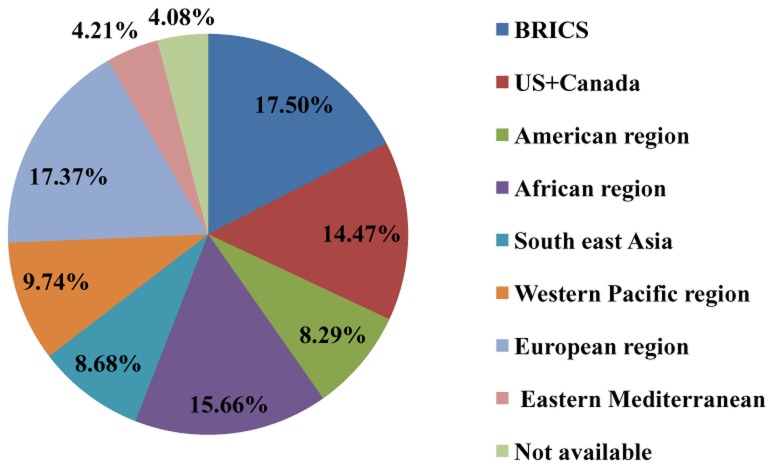
Distribution of coordinator centres of clinical trials for NDs. BRICS: Brazil, Russia, India, China and South Africa, N.A: not available.

The last topic we have investigated is the location of the trials coordinator. Less affluent countries, and particularly emerging ones (including BRICS - Brazil, Russia, India, China and South Africa), may be a target for trial location of the trials coordinator because most NDs are endemic to these countries. Additionally, some of these countries have developed a capable scientific community and their pharmaceutical market is growing faster than in affluent countries. Lower costs may be another reason for locating trials in less affluent countries, even if cost is not the most important driver of trials location of the trials coordinator [14] [15]. Coordinator centres of trials on NDs are distributed among OECD countries (34.7%), BRICS (17.5%) and other less affluent countries (43.7%) (in 4.1% of trials the coordinator centre is unspecified). Figure 4 shows the distribution of coordinator centres using country clusters adopted by the WHO classification (http://www.who.int/about/regions/en/index.html, last access, 15^th^ of February 2013), but considering separately the BRICS group. BRICS together take first place, with India playing a leading role (9.3% of trials). The European Region accounts for 17.4% of trials, with 7.1% in the major EU-5 countries. The US and Canada account for 14.5% of trials. In other regions, Bangladesh (3.8%) and Thailand (3,7%) are the countries more involved in trials for NDs. Other countries are all below 3%.

## Discussion

Our research has confirmed the growing interest in NDs of previous analyses, with an important increase in the number of trials in 2011–12.

All NDs considered show at least one interventional clinical trial, with few exceptions, including Dracunculiasis (Guinea-worm disease), food-borne Trematodiases and Myiasis. Additionally, we found a large number of studies in which patients' enrolment has been completed (57.8% of studies) and 28% completed was in in phase III. Hence, the present pipeline is the result of an investment that started several years ago. Prevention and treatment are the objectives of 49.3% and 43.2% of studies, respectively, whereas basic science and diagnosis/screening technologies are disregarded. Prevention is the main target in virus-related diseases, whereas for non-virus related diseases, with the relevant exception of Shigella, treatment is the main target.

The research has been focusing on Leishmaniasis, Dengue, Rabies, Salmonella and Cholera. Other diseases, such as Fascioliasis, Relapsing Fever, Giardiasis, Amebiasis, Echinococcosis or Yaws, have less than four trials each.

The target choice may have different drivers.

It seems that the prevalence of the disease is not the main driver of the research target, e.g. trials for Soil-transmitted Helminthiasis (Ascariasis and Trichuriasis), which show a high prevalence, are rare, with the exception of Hookworm infection.

The unmet need may be another driver. However, the evidence is rather controversial, and there are many cases not supporting this hypothesis. For example, Buruli Ulcer is still considered a ND, but it may be easily managed using a combination of antibiotics, if diagnosed early. Only four trials have been found for Buruli Ulcer between 2005 and 2012, and all of them were focused on clarithromycin and not on early diagnosis issues. Another example is Leishmaniasis, where the primary object of most trials (17 in phase III and 10 in phase IV) is to test the efficacy of drugs that are already approved and included in the WHO recommendation, whereas only one trial focuses on vaccines.

The third driver may be the risk of disease diffusion. In fact, many trials were found for NDs with a high risk of diffusion due to their viral nature, including tick-born and Japanese encephalitis (Arbovirus infection group), Dengue, Rabies and Salmonella.

The role of pharmaceutical companies in directly sponsoring clinical research for NDs is rather limited and is very concentrated in a few companies (Sanofi Aventis, Novartis, and Intercell AG), with a focus on Arbovirus infection and Dengue. Research centres and public institutions are much more involved, whereas Foundations and NGOs play a minor role.

Whereas sponsors are either international organisations or concentrated in the US, BRICS are increasing their role in location of coordinator centres. This may be motivated by the higher prevalence of NDs in these countries, their emerging economies and the increasing research standards guaranteed by trials sites.

The present study has some limitations: (i) not all trial databases were scrutinised, even if some of the excluded ones show a very low potential contribution to the dataset; (ii) databases are not complete for some topics: e.g., trial phase was unspecified for 30% of trials; and (iii) the analysis is purely descriptive, even if some relationships have been qualitatively investigated.

Despite its limitations, this study has for many aspects integrated the evidence on R&D in NDs and updated this evidence on 2012. Future contributions may further investigate trials metrics, such as the formulation, setting of administration and length of treatment, to understand their consistency with low-income countries' characteristics [5], and the possible explicative variables of location, target (disease) and object (basic science, prevention, diagnosis/screening, treatment).

## References

[1] Molyneux DH (2010) Neglected diseases, but unrecognized successes- challenges and opportunities for infectious disease control. Lancet 364: 380: 83. 10.1016/S0140-6736(04)16728-7 15276399

[2] Hotez PJ, Molyneux DH, Fenwick A, et al (2007) Control of Neglected Tropical Diseases. N Eng J Med 357: 1018–27. 10.1056/NEJMra064142 17804846

[3] WHO Neglected tropical diseases. http://www.who.int/neglected_diseases/diseases/en/. Accessed 20 December 2012.

[4] Public Library of Science Neglected Tropical Diseases Journal Scope. http://www.plosntds.org/static/scope. Accessed 20 December 2012.

[5] LSE and Wellcome Trust (2005) The new landscape of neglected disease drug development. Pharmaceutical R&D Policy Project Available: http://www.wellcome.ac.uk. Accessed 15 November 2012.

[6] Liese B, Rosenberg M, Schratz A (2010) Programmes, partnerships, and governance for elimination and control of neglected tropical diseases. Lancet 375: 67–76. 20109865 10.1016/S0140-6736(09)61749-9

[7] Reich MR Ed (2002), Public-Private Partnerships for Public Health. Harvard Series on Population and International Health. Harvard Center for Population and Development Studies, Cambridge

[8] Moran, et al (2009) Neglected Disease Research and Development: How Much Are We Really Spending? PLoS Med 6: e1000030. 10.1371/journal.pmed.1000030 PMC2634791 19192946

[9] WHO (2007). Global plan to combat neglected tropical diseases 2008–2015. Geneva: World Health Organization.

[10] Uniting to Combat NTDs, The London Declaration. http://unitingtocombatntds.org/downloads/press/london_declaration_on_ntds.pdf, Accessed 20 December 2012.

[11] Moran M, Guzmanj, Abela-Oversteegen L, et al (2011) Neglected disease research and development: Is innovation under threat? Policy Cures Report. Available: http://policycures.org/downloads/g-fi nder_2011.pdf. Accessed 24 April 2014.

[12] BIO Ventures for Global Health Developing new drugs and vaccines for neglected diseases of the poor: the product developer landscape. Avaliable http://www.bvgh.org/LinkClick.aspx?fileticket=h6a0cJK9drg%3d&tabid=91. Accessed 24 April 2014.

[13] Pedrique B, Strub-Wourgaft N, Some C, et al (2013) The drug and vaccine landscape for neglected diseases (2000–11): a systematic assessment. The Lancet Global Health 1: 371–379. 10.1016/S2214-109X(13)70078-0 25104602

[14] Jommi C, Paruzzolo S (2007) Public Administration and R&D localisation by pharmaceutical and biotech companies: a theoretical framework and the Italian case study. Health Policy 81: 117–130. 16824641 10.1016/j.healthpol.2006.05.010

[15] Gehring M, Taylor Rod S, Mellody Marie, et al (2013) Factors influencing clinical trial site selection in Europe: the Survey of Attitudes towards Trial sites in Europe (the SAT-EU Study),. BMJ Open 3: e002957. 10.1136/bmjopen-2013-002957 PMC3831096 24240138

[16] Hotez P (2008) Forgotten People, Forgotten Diseases, Washington, DC: ASM Press.

[17] WHO Neglected tropical diseases: Foodborne trematode infections. http://www.who.int/foodborne_trematode_infections/en/. Accessed 31 December 2012.

[18] WHO Neglected tropical diseases: Rabies. http://www.who.int/rabies/en/index.html. Accessed 31 December 2012.

[19] Weekly epidemiological record Relevé épidémiologique hebdomadaire (2007) 82^nd^ year: 49/50:. 82: 425–436 Accessed 31 December 2012.

[20] WHO Neglected tropical diseases: Cysticercosis/Taeniasis. http://www.who.int/neglected_diseases/diseases/cysticercosis_symptoms/en/index.html. Accessed 31 December 2012.

[21] WHO Neglected tropical diseases: Yaws. http://www.who.int/mediacentre/factsheets/fs316/en/index.html. Accessed 31 December 2012.

